# Let’s Not Waste Time: Using Temporal Information in Clustered Activity Estimation with Spatial Adjacency Restrictions (CAESAR) for Parcellating FMRI Data

**DOI:** 10.1371/journal.pone.0164703

**Published:** 2016-12-09

**Authors:** Ronald J. Janssen, Pasi Jylänki, Marcel A. J. van Gerven

**Affiliations:** Radboud University, Donders Centre for Brain Cognition and Behaviour, Nijmegen, the Netherlands; Hangzhou Normal University, CHINA

## Abstract

We have proposed a Bayesian approach for functional parcellation of whole-brain FMRI measurements which we call Clustered Activity Estimation with Spatial Adjacency Restrictions (CAESAR). We use distance-dependent Chinese restaurant processes (dd-CRPs) to define a flexible prior which partitions the voxel measurements into clusters whose number and shapes are unknown a priori. With dd-CRPs we can conveniently implement spatial constraints to ensure that our parcellations remain spatially contiguous and thereby physiologically meaningful. In the present work, we extend CAESAR by using Gaussian process (GP) priors to model the temporally smooth haemodynamic signals that give rise to the measured FMRI data. A challenge for GP inference in our setting is the cubic scaling with respect to the number of time points, which can become computationally prohibitive with FMRI measurements, potentially consisting of long time series. As a solution we describe an efficient implementation that is practically as fast as the corresponding time-independent non-GP model with typically-sized FMRI data sets. We also employ a population Monte-Carlo algorithm that can significantly speed up convergence compared to traditional single-chain methods. First we illustrate the benefits of CAESAR and the GP priors with simulated experiments. Next, we demonstrate our approach by parcellating resting state FMRI data measured from twenty participants as taken from the Human Connectome Project data repository. Results show that CAESAR affords highly robust and scalable whole-brain clustering of FMRI timecourses.

## Introduction

The brain is generally assumed to consist of interconnected functional modules. This principle takes central stage in connectomics research, referring to the study of the properties of these connection patterns [1]. Hence, connectomics presupposes some definition of nodes to be connected. This node definition can be linked to different scales, ranging from single neurons to brain regions. To a large extent, the scale of node definition is dictated by the measurement method employed to probe network architecture. In the case of functional magnetic resonance imaging (FMRI), the smallest accessible scale is given by the voxel size.

Given the large numbers of voxels in whole-brain analyses, it is usually more convenient to group voxels into functionally coherent regions. This begs the question of how to accomplish this. The simplest approach is to use a predefined atlas, warped to individual participants’ brains [2]. A better approach is to parcellate the brain based on functional signals. This way, regions are formed that represent functionally coherent modules, which is important for subsequent functional analyses [3]. A number of approaches have been suggested for clustering FMRI data, including K-means [4], hierarchical clustering [5], spectral clustering [6–8], boundary based segmentation [9] and more (e.g. [10–13]).

Generally, extant parcellation approaches require the user to select the number of clusters in the parcellation. Non-parametric Bayesian clustering approaches, like those presented in [11–13], are one way of remedying this issue by estimating the number of clusters along with the parcellation. We recently showed that such an approach can be used to provide a robust, meaningful parcellation of the striatum [13].

A common tactic in parcellation approaches is to base the parcellation on the voxel-wise functional connectivity, usually measured with the Pearson correlation coefficient. The main advantage of this approach is one of scaling. Such methods can operate on a group-average correlation-matrix, hence they scale independent of the number of timepoints and participants. The disadvantage is that they do not model the cluster timecourses directly, hence they do not provide an estimate of these. This is usually solved by going back to the data and computing a mean timecourse.

A more elegant approach would be to include this estimation in the model formulation, this way one need only apply the model once and the output would consist of both a set of clusters and their corresponding timecourses. Such a formulation also allows the incorporation of assumptions on the cluster timecourses. Given that the blood-oxygen-level dependent (BOLD) signal is assumed to represent neuronal signal after convolution with the haemodynamic response function [14], incorporating a smoothness assumption should improve the estimation of cluster time courses and, through this, the parcellation. The approach presented in [15] is an example of a model that incorporates temporal assumptions about time courses. The model aims to decompose the data in a set of spatial maps and associated time courses, similar to principal/independent component analysis (PCA and ICA respectively). However, like ICA, this is not strictly a parcellation approach, because the resulting components can have spatial overlap as well as negative weights.

In this paper, the model presented in [13], which we call Clustered Activity Estimation with Spatial Adjacency Restrictions (CAESAR), is extended to include assumptions about temporal smoothness. This is achieved by assuming a Gaussian process prior to model the temporally smooth haemodynamic signals that give rise to the measured FMRI data. We addressed the computational challenges that emerge from this extension and show that the resulting approach allows efficient and robust estimation of whole-brain parcellations from FMRI timecourses.

## Materials and Methods

**Fig 1:**
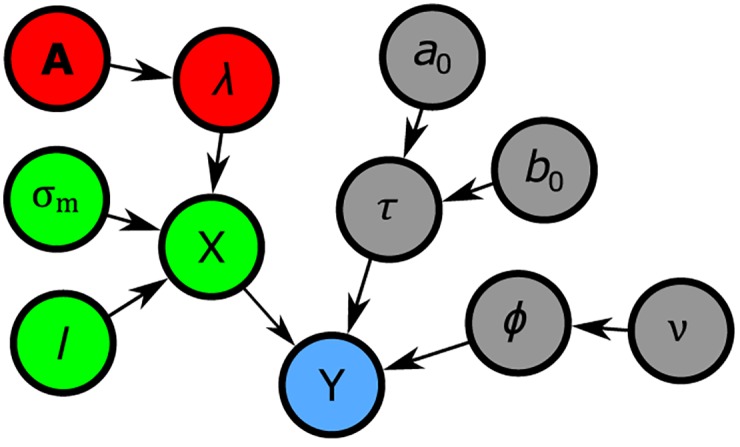
Graphical representation of the model. **Y** denotes the observed data, the red nodes represent the clustering prior, green nodes form the Gaussian process model and gray nodes designate variables in the noise model. Put together, **X**, **Y** and the noise model form the observation model.

We proceed by describing the different building blocks of CAESAR, as summarized in the graphical model shown in Fig 1. For the sake of consistency, we use ‘nodes’ to refer to the elements being clustered, be they voxels or mesh nodes (for volumetric and surface-mapped data respectively). A Matlab implementation of CAESAR is provided in S1 Code and maintained at https://github.com/ccnlab/ddCRP.

We are interested in the posterior distribution

$$\begin{array}{c} p\left(\pi |\text{Y},\theta \right)=Z^{-1}\underset{\text{observation}\;\text{model}}{\underset{︸}{p(\text{Y}|\text{X},\pi ,\theta )}}\overset{\text{timecourse}\;\text{prior}}{\overset{︷}{p(\text{X}|\pi ,\theta )}}\underset{\text{parcellation}\;\text{prior}}{\underset{︸}{p(\pi |\theta )}}, \end{array}$$ (1)

where **π** represents a parcellation, ***θ*** denotes the hyperparameters, **Y** is the data and *Z* = *p*(**Y**|***θ***) is the normalization term. Our model consists of three components: the observation model, the timecourse prior and the parcellation prior. In short, the observation model encodes the assumption that FMRI data are noisy observations of the underlying cluster timecourses. These timecourses are modelled in the timecourse prior as a Gaussian Process (GP) with a smoothness-promoting covariance function, in order to describe autocorrelations in BOLD fluctuation. The parcellations are drawn from a non-parametric prior that allows us infer the number of clusters as well as enforce spatial contiguity of those clusters. The following sections describe each of these components in more detail.

### Observation model

Let us assume that we have collected a *N* × *T* FMRI data matrix **Y**, where *N* is the number of nodes and *T* the number of time points. Given a partitioning **π** = [*π*_1_, …, *π*_*N*_]^T^ of the nodes into *K* clusters, and a *K* × *T* matrix **X** of unobserved cluster timecourses, we model the observed data as

$$\begin{array}{c} p\left(\text{Y}|\text{X},\phi ,\tau ,\pi \right)=\prod_{n=1}^{N}\prod_{t=1}^{T}N\left(y_{n,t}|x_{\pi_{n},t},{\left(\tau \phi_{t}\right)}^{-1}\right), \end{array}$$ (2)

where *π*_*n*_ ∈ {1, 2, …, *K*} indicates the cluster assignment of node *n*, *τ* is an overall noise precision parameter, and *ϕ*_*t*_ models time-specific deviations from the overall noise level caused, e.g., by measurement errors or other confounds. We implement an outlier-robust Student-*t* observation model by assigning independent gamma priors 2*ϕ*_*t*_ ∼ Gamma(*ν*/2, *ν*/2) to the time-specific noise precisions and fixing the degrees of freedom parameter to *ν* = 4. Assuming the observations are normalized to unit variance, we choose an uninformative prior for *τ* by setting *τ* ∼ Gamma(*a*_0_, *b*_0_) with *a*_0_ = 1 and *b*_0_ = 0.01.

When analysing multiple datasets, the observation model is simply the product of Eq (2) over datasets with fixed **π**.

### Cluster timecourse prior

The observed FMRI timecourses are known to be generated by smooth and relatively slowly varying haemodynamic signals that are confounded by more broadly distributed noise during the measurement process [16]. To incorporate this background knowledge into our model, we first rewrite the cluster timecourses from Eq (2) as *x*_*k*,*t*_ = *x*_*k*_(*t*) to emphasize that we are constructing a prior for functions of time. Next, we construct priors *p*(*x*_*k*_(*t*)) that promote smooth, slowly varying cluster timecourses, because they are used in the observation model Eq (2) to group together node signals that are generated by similar underlying signals. To this end, we place independent, smoothness-promoting Gaussian-process priors on the unobserved cluster-timecourses.

A standard zero-mean GP, denoted by $x_{k}\left(t\right)∼\mathrm{GP}\left(0,\kappa \left(t,t^{\prime }\right)\right)$, is defined by choosing a suitable covariance function *κ*(*t*, *t*′) = Cov(*x*_*k*_(*t*), *x*_*k*_(*t*′)) that encodes our prior assumptions on the smoothness properties of the unknown function *x*_*k*_(*t*) [17]. For *T* unobserved function values **x**_*k*_ = [*x*_*k*_(*t*_1_), …, *x*_*k*_(*t*_*T*_)]^T^ associated with time points *t*_1_, …, *t*_*T*_, this formulation results in a *T*-dimensional multivariate normal prior distribution for each **x**_*k*_:

$$\begin{array}{c} p\left({\text{x}}_{k}|\psi \right)=N\left(0,\text{K}\right), \end{array}$$ (3)

where the *T* × *T* covariance matrix **K** defines the prior covariances between each component pair of **x**_*k*_: **K**_*t*,*t*′_ = Cov(*x*_*k*_(*t*), *x*_*k*_(*t*′)) = *κ*(*t*, *t*′). The vector ***ψ*** contains the hyperparameters of the covariance function that control the overall scale and smoothness properties of the unknown function *x*_*k*_(*t*).

For our clustering model, we choose the Matérn class covariance function which is given by

$$\begin{array}{c} \kappa \left(t,t^{\prime }|\psi \right)=\kappa_{\nu }\left(r\right)=\sigma_{m}^{2}\frac{2^{1-\nu }}{\Gamma (\nu )}{\left(\eta r\right)}^{\nu }K_{\nu }\left(\eta r\right), \end{array}$$ (4)

where *r* = *t* − *t*′, $\eta =\sqrt{2\nu }/l$ and $K_{\nu }$ is a modified Bessel function of order *ν*. We denote the free GP hyperparameters with $\psi =\{\sigma_{m}^{2},l\}$, where $\sigma_{m}^{2}$ is the magnitude parameter that controls the overall prior scale (or variance) of **x**_*k*_, and $l=\sqrt{2\nu }/\eta$ is the characteristic length-scale parameter that controls how rapidly **x**_*k*_ can vary with respect to *t*: the smaller *l*, the faster **x**_*k*_ can vary.

Different Matérn-class priors are obtained by adjusting *ν*: the larger the value, the stronger the smoothness assumption (for details, see [17]). We set *ν* = 3/2, which results in a stochastic process that can be represented as a second-order stochastic differential equation [18]. This process is smooth, yet is not overly conservative with respect to that property. Note that if we instead choose $\kappa \left(t,t^{\prime }|\psi \right)=\sigma_{m}^{2}\delta \left(r\right)$, where *δ*(*r*) = 1 if *r* = 0 and *δ*(*r*) = 0 otherwise, the temporally-independent model of [13] is recovered.

We set $\sigma_{m}^{2}=0.1$ to reflect our assumption of a SNR of 0.1/0.9, whereas *l* = 3.6 approximates the autocorrelation of the default haemodynamic response function (HRF) provided in the SPM software package (SPM8; http://www.fil.ion.ucl.ac.uk/spm/).

### Cluster assignment prior

The Chinese restaurant process (CRP) is a commonly used construction to implement Dirichlet process mixture priors for random cluster partitions (see, e.g. [19, 20]. The traditional CRP forms a prior for random partitions, *p*(**π**), by sequentially assigning *π*_*n*_ to one of the existing clusters or to a new cluster for each *n* = 1, …, *N* conditioned on the previous assignments **π**_1:*n*−1_. Regular Gibbs sampling with the CRP prior attempts to update each *π*_*n*_ separately conditioned on **π**_−*n*_, which often results in slow convergence especially with large *N* (see, e.g., [21]). Improving convergence or incorporating spatial constraints would require additional split-merge updates with sequential allocation. Instead of these modifications, we use an alternative construction known as the distance-dependent CRP (dd-CRP) that automatically implements split-merge steps via regular Gibbs sampling and allows straightforward and flexible definition of constraints that ensure spatially connected partitions [13, 21].

In contrast to the regular CRP which works directly with cluster assignments *π*_*n*_, the dd-CRP prior associates each node *n* with exactly one other node *m* by generating a link λ_*n*_ = *m* from node *n* to *m* with probability

$$\begin{array}{c} p\left(\lambda_{n}=m|\text{D}\right)\propto f\left(d_{n,m}\right), \end{array}$$ (5)

where λ_*n*_ ∈ {1, 2, …, *N*}, matrix **D** contains some appropriate distance measures *d*_*n*,*m*_ = [**D**]_*n*,*m*_ between nodes *n* and *m*, and *f*(*d*) is a non-increasing decay function that satisfies *f*(*d*) ≥ 0 and *f*(∞) = 0 [21]. The key difference to regular CRPs is that the prior probability of λ_*n*_ depends only on the distance measures **D** and not on the cluster assignments **π**. The partition **π**(**λ**) is formed indirectly by the links **λ**. That is, all the nodes that are interconnected via their link assignments form a cluster.

In the case of volumetric FMRI-data, *d*_*n*,*m*_ could be set to the Euclidean distance between the midpoints of voxels *n* and *m*. When dealing with surface-mapped data, one would ideally use the geodesic between nodes *n* and *m* to determine the distance. A convenient approximation of this is the shortest path length between nodes in the surface mesh. We define the decay function such that nodes can only connect to their immediate neighbours, which, in the case of surface-mapped data, corresponds to

$$\begin{array}{c} f\left(d\right)=\{\begin{array}{cc} 1 & \text{if}\;d\leq 1\\ 0 & \text{otherwise}. \end{array} \end{array}$$ (6)

This results in a neighbourhood of at most six possible link assignments for each node in a mesh. If we collect all the weighted distances into a sparse *N* × *N* matrix **A** so that **A**_*n*,*m*_ = *f*(*d*_*n*,*m*_), we can write our prior for the links as

$$\begin{array}{ccc} p(\lambda |\text{A}) & = & \prod_{n=1}^{N}p\left(\lambda_{n}|\text{A}\right)\\ & = & \prod_{n=1}^{N}\prod_{m=1}^{N}{\left(\frac{A_{n,m}}{\sum_{i=1}^{N}A_{n,i}}\right)}^{\left[\lambda_{n}=m\right]} \end{array}$$ (7)

with [⋅] being the Iverson bracket. This prior formulation enables convenient implementation of various distance weighting schemes and spatial constraints.

In Eq (5) we assume that *d*_*n*,*n*_ = 0 and that *f*(0) defines the probability that node *n* links to itself. This corresponds to the concentration parameter in a traditional CRP, which controls the probability of starting new clusters. Note that in the ddCRP, having λ_*n*_ = *n* does not necessarily put that node in a singleton cluster, as other nodes might still be linked to it. As such, the influence of the value chosen for *f*(0) is limited towards encouraging smaller parcels in the case of large values. This is perhaps best illustrated by considering the most extreme settings for this parameter.

Suppose the parameter is set to infinity, then nodes will almost surely all link to themselves, resulting in only singleton clusters. Hence, large values encourage smaller clusters, as in a traditional CRP. In contrast, if the parameter is set to zero in a CRP, we would almost surely get one cluster containing all nodes, whereas the ddCRP is free to make any partition with the constraint that clusters contain at least two nodes. This is due to the fact that if we represent customer links as a graph, the number of clusters is defined by the number of cycles and the smallest possible cycle without self-linking is a two cycle.

Certain questions might be best resolved at a certain scale, hence it might be desirable to be able to provide stricter constraints on the scale of the clustering. Therefore, we introduce an improper prior on cluster size. We chose to constrain size rather than number of clusters in order to obtain clusters with comparable sizes. This is achieved by multiplying Eq (1) with

$$\begin{array}{c} p\left(\text{s}|d,w\right)=\prod_{k=1}^{K}\{\begin{array}{cc} \exp(-\frac{{\left(d-s_{k}|\right)}^{2}}{2w^{2}}) & \text{if}\;s_{k}<d\\ 1 & \text{otherwise}, \end{array} \end{array}$$

where **s** = [*s*_1_, *s*_2_, ⋯, *s*_*K*_] is the vector of cluster sizes, *d* is a lower bound on the cluster sizes and *w* controls the strength of this constraint.

### Bayesian Posterior Inference

We use Markov chain Monte Carlo (MCMC) methods to obtain samples from Eq (1). The main Gibbs sampling procedure is described in S1 Inference, together with an additional population Monte Carlo framework that can be used to run multiple Gibbs chains in parallel and to combine them after each iteration to speed up convergence.

The most time consuming part of the sampling procedure is the Gibbs sampling run over the link assignments λ_1_, …, λ_*N*_ using the conditional posterior

$$\begin{array}{c} p\left(\lambda_{i}|\lambda_{-i},\text{Y},\theta ,\text{A}\right)\propto p\left(\text{Y}|\lambda ,\theta \right)p\left(\lambda_{i}|\text{A}\right)\;, \end{array}$$

where the *K* × *T* dimensional latent variable **X** is integrated out to obtain the marginal likelihood: *p*(**Y**|**λ**,***θ***) = ∫*p*(**Y**|**X**, **λ**, ***θ***)*p*(**X**|**λ**, ***θ***)*d* **X**. This averaging over **X** is essential for an efficiently converging sampling procedure since the dimension of **X** changes constantly as clusters are being split apart and merged together. Integrations over **X** scale as $O(T^{3})$, because the size of the multivariate GP prior covariance **K** defined in Eq (3) increases with the number of observations *T*. In practice, this can become prohibitively expensive since typical FMRI datasets can contain thousands of time points.

In S1 Inference we also describe a batch method for conditional inference on **X** which assumes that the hyperparameters ***ψ*** remain fixed during the Gibbs sampling of the links **λ** and computes all $O(T^{3})$ scaling matrix operations only when the hyperparameters are changed. The batch method is most efficient for data sets with roughly *T* < 10000.

Alternatively, if *T* is very large, one can transform the GP prior Eq (3) into an equivalent state-space form as described by [18]. Using the resulting linear dynamical system, the marginal likelihood and the conditional posterior of **X** can be computed by Kalman filtering and smoothing, which scales linearly in *T*. However, with our implementations and data sets, the batch method was at least an order of magnitude faster compared to the filtering approach. Hence, in the following we report only the results obtained with the batch approach.

### Experiments

The clustering model was validated using simulated experiments and subsequently applied to resting state FMRI (rsFMRI) data for empirical validation.

#### Simulation study

To validate CAESAR and the GP-extension, we simulated realistic FMRI data from a spatially constrained cluster structure.

**Fig 2:**
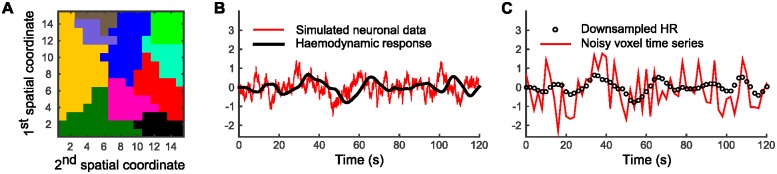
Illustration of the simulation procedure. Panel A: Generate a random partition for a two-dimensional grid using spatial constraints. Panel B: Generate a neuronal timecourse (sampled at 200 Hz) for each cluster and filter it using the canonical haemodynamic response function. Panel C: Down sample the HR signal to 0.5 Hz and draw the node timecourse by adding Gaussian noise according to the desired signal-to-noise ratio.

Fig 2 illustrate the simulation process. First, the number of clusters *K* was fixed to some desired value and then the nodes of a two-dimensional 15 × 15 grid were randomly partitioned into *K* clusters by setting the cutoff distance in the dd-CRP decay function Eq (6) to 1 pixel, which allows within-cluster connections only to the four nearest neighbours for each node. Fig 2A illustrates a resulting partition with *K* = 10. The actual clusters were generated by simple region growing using *K* randomly selected starting nodes meaning that the partition was not generated from a dd-CRP prior. This way we can verify that the modelling framework can learn general partitions following some known distance constraints.

For each cluster, the node measurements were simulated by first generating a neuronal timecourse with a sampling frequency of 200 Hz, indicated by the red line in Fig 2B that represents the unobserved local field potentials associated with each functional cluster. These timecourses were drawn from a Matérn-class GP prior with hyperparameters *ν* = 1/2, $\sigma_{m}^{2}=1$, and *l* = 2, which corresponds to an Ornstein-Uhlenbeck process with mean reversion rate $\eta =\sqrt{2\nu }/l=0.5$. The spectral density of the process decays proportional to 1/(*η*^2^ + (2*πf*)^2^), which makes it a reasonable approximation for synaptic activity [22]. The state variable $\tilde{x}\left(t\right)$ of the equivalent stochastic differential equation representation of the process is one-dimensional corresponding to a first order autoregressive model, and the transition density is given by $p\left(\tilde{x}\left(t+\Delta t\right)|\tilde{x}\left(t\right)\right)=N\left(F_{t}\tilde{x}\left(t\right),Q_{t}\right)$, where *F*_*t*_ = exp(−*η*Δ*t*), $Q_{t}=\sigma_{m}^{2}\left(1-\exp\left(-2\eta \Delta t\right)\right)$ and Δ*t* = 1/200 s (for details, see [18]). From this simulated neuronal signal, BOLD signals were obtained by filtering the neuronal signal with the canonical haemodynamic response function, as indicated by the black line in Fig 2B. Finally, simulated FMRI measurements (the red line in Fig 2C) were formed by down sampling the BOLD signal to 0.5 Hz (black circles in Fig 2C) and adding independent Gaussian noise. The variance of this noise was adjusted according to the desired signal-to-noise ratio (0.1/0.9 in Fig 2C).

In our experiments, we compare the dd-CRP solution using either a temporally independent Gaussian timecourse prior, $p\left({\text{x}}_{k}|\sigma_{m}^{2}\right)=N\left(0,\sigma_{m}^{2}{\text{I}}_{T}\right)$ (IT-model), or a temporal Matérn-class GP prior defined by Eqs (3) and (4). As using the GP-model amounts to low-pass temporal-filtering (a common preprocessing step) we also apply the IT-model to data that was low-pass filtered with a 0.1 Hz cut-off. We set $\sigma_{m}^{2}=1$ for the IT-model. Our preliminary results indicated that the GP-based model was not found to be sensitive to the hyperparameter values but with the independent model the number of estimated clusters was found to vary more with different values of $\sigma_{m}^{2}$. In addition, we compared both variants of CAESAR to the one proposed by Baldassano, Beck and Fei-Fei [11], which was applied to the correlation matrix of the filtered data. Parameter settings were as follows: *α* = 1, *μ*_0_ = 0, *κ*_0_ = 0.0001, *ν*_0_ = 1, $\sigma_{0}^{2}=0.1$. Parameters settings for *μ*_0_, *κ*_0_ and *ν*_0_ correspond to those suggested by the authors. The concentration parameter *α* was set to match our choice in prior. As the authors did not mention a principled way of choosing $\sigma_{0}^{2}$, it was tuned for optimal performance on a dataset with *N* = 625 nodes, *K* = 20 clusters, *T* = 15 min of data and a SNR of 0.1/0.9.

Because the Gibbs sampler for the dd-CRP was found to converge quickly with all simulated data sets that have a true underlying cluster structure, the population Monte Carlo algorithm from S1 Inference was not required for the simulated experiments of this section. With both priors, the sampling was done using the same random number sequence and the same randomly initialized partition with 20 clusters. The first 50 samples were discarded as burn-in and the co-assignment matrices, whose non-zero elements indicate that two nodes are assigned to the same cluster, were estimated as the mean of the co-assignments of the next 100 samples. To obtain the final cluster timecourse estimates, first a fixed partition was generated by joining together nodes whose mean reassignment exceeded 0.9, and then another 50 samples were taken for the cluster timecourses and all the hyperparameters with that fixed partition.

#### FMRI data

To empirically validate CAESAR as a whole-brain parcellation approach, rsFMRI datasets for 38 participants (the 40 unrelated participants set) were obtained from the Human Connectome Project (HCP) database [23]. Each dataset consists of four runs of 15-minute rsFMRI recordings. A complete description of data acquisition, including informed consent and ethical approval, and preprocessing steps has been reported elsewhere [24, 25]. Briefly, task-free FMRI data was acquired with 2 mm isotropic voxels and a repetition time (TR) of 0.72 s. Both T1- and T2-weighted images were used to reconstruct the cortical surface and these were registered to the Conte69 cortical-surface [26]. Functional data was mapped to the participant’s cortical surface and transformed from there to the Conte69 surface.

With such a short TR, modelling the temporal dependencies is especially useful as it allows the model to characterise the measurement noise more accurately. Using surface-mapped data also simplifies the computations for a ddCRP model as the node neighbourhood is generally smaller than in a volume representation. Moreover, it also precludes direct connections between opposite banks of a sulcus, which would be considerably more difficult to exclude otherwise.

Datasets were split into two groups, the first 20 participants forming the first group and the remainder in the second group. For each of the groups we examined runs 1 and 2, for a total of four group-level datasets. Analyses were restricted to the first 250 data points (3 min) from each participant in order to reduce computational time. In addition, we performed this analysis both with and without the cluster-size prior. The parameters for the size prior were *d* = 200 and *w* = 5. This soft bound results in a manageable number of clusters, while still allowing the model some freedom in determining cluster sizes and, through this, the number of clusters.

Due the size and richness of the FMRI datasets, the posterior landscape is difficult to explore with single chain MCMC. For this reason, posterior inference was performed using a population MC approach [27]. In short, the approach consists of the following steps:

1. Initialize *J* MCMC chains.
2. Take *N* steps for all chains save the final state as sample
3. Randomly pick samples (with replacement) from these samples to reinitialize the *J* chains.
4. Repeat steps 2 and 3 until sufficient samples have been obtained.

The probability of selecting the *j*th sample to reinitialize a chain is proportional to the importance weight of that sample, which is proportional to its posterior probability divided by the product of transition densities for all update steps since the last reinitialization. The underlying idea is that, at each step, the chains can search the local space independent of each other and the algorithm uses the best of these to start the next search. A full description of our implementation is given in S1 Inference.

We used 50 parallel chains, with each chain performing one sweep over hyperparameters and 11 sweeps over the link assignments before resampling. The first link-assignment sweep was done at a temperature of 1000 (i.e. all log assignment-probabilities were multiplied by 0.001) to encourage exploration of the search space, the remainder of the sweeps were done with a temperature of one. We collected 100 samples for both hemispheres and took the sample with the highest posterior probability as an approximation to the maximum a-posteriori solution.

In order to examine CAESAR’s performance on empirical data, we compared reproducibility and explained variance with spatially-constrained Ward-clustering on low-pass-filtered data (0.1Hz cut-off). The reason we stray from the comparison with the connectivity-based model is due to their running times when applied to the ≈30K nodes in a hemisphere. We chose spatially-constrained Ward-clustering as it was found to be the best among several commonly used approaches [28]. Ward-clustering starts with only singleton clusters and iteratively merges the two clusters with the lowest squared Euclidean distance between them. Clusters were merged until the number of clusters matched the corresponding result from CAESAR.

## Results

In this section we will first describe CAESAR performance on simulated data, followed by results obtained with rsFMRI data. In comparisons between temporally-independent and temporally-dependent priors, we will refer to these as IT- and GP-model respectively.

### Simulations

Accuracy, robustness and efficiency of the GP-model variant of CAESAR were examined and compared to that of the IT-model as well as the connectivity-based model from [11] to assess performance. While there are a large number of alternative approaches to compare with, we chose to limit ourselves to this model because it is closely related to CAESAR and it outperforms the alternatives.

#### Accuracy

**Fig 3:**
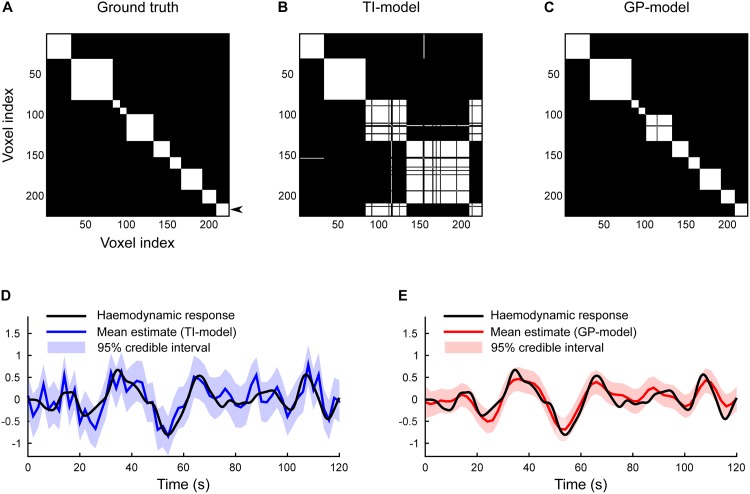
Comparison of timecourse priors in CAESAR. Temporally-independent Gaussian prior (IT-model) is compared with a Matérn-class GP prior (GP-model) using simulated data from Fig 2 with attributes *N* = 225, *K* = 10, and SNR = 0.1/0.9. The true partition is shown in Fig 2A and the corresponding co-assignment matrix is shown here in panel A. Panels B and C show the respective posterior mean estimates using the IT-model and the GP-model. Note that while neither model shows uncertainty in their parcellation estimate, the GP-model is superior in recovering the true cluster structure. Illustrated in panels D and E are the posterior mean and 95% credible interval estimates of the cluster timecourse from an example cluster, which is indicated with an arrow in panel A. The true cluster assignment was used here to specifically illustrate the difference in timecourse recovery.

Fig 3 shows an illustrative experiment with a 15 × 15 grid corresponding to *N* = 225 nodes, *K* = 10 clusters, and SNR equal to 0.1/0.9. The true cluster structure is shown in Fig 2A and the corresponding true co-assignment matrix in Fig 3A. The mean co-assignments with TI- and GP-models are shown in Fig 3B and 3C. The IT-model recovers only 4 clusters merging together all the smaller ones with their neighbours. The GP-model recovers 12 clusters, which, aside from placing two individual nodes in their own singleton clusters, corresponds to the ground truth. Neither model shows any uncertainty in their estimation.

Fig 3D and 3E show the cluster timecourses estimates for the HR simulated in Fig 2B and the corresponding cluster is indicated with an arrow in Fig 3A. In order to isolate the effects of choice in prior on timecourse recovery, the true cluster assignments were used for both models. With the IT-model, the timecourse estimate is clearly not smooth because of the confounding effects of the observation noise. Timecourse estimates deteriorate as the SNR or the number of nodes in a cluster decrease. In contrast, the GP-model’s estimate is smooth and the marginal 95% credible interval includes the true cluster timecourse. This example cluster includes only 16 nodes which makes it harder to estimate the exact timecourse. With the larger clusters consisting of roughly 30 nodes or more, almost perfect reconstruction can be recovered. This example clearly shows that more accurate cluster reconstructions can be obtained by incorporating prior knowledge about the smoothness of the cluster timecourse.

#### Robustness

**Fig 4:**
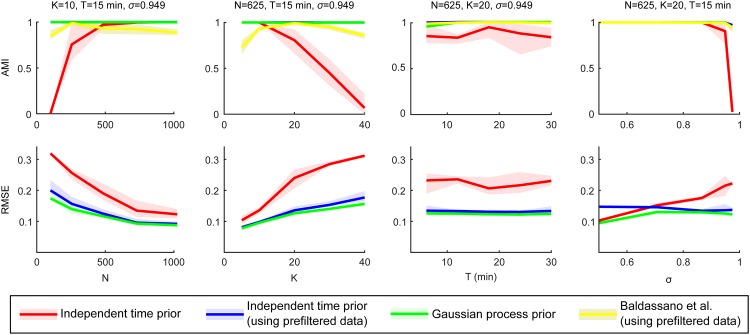
Accuracy of dd-CRP based methods using simulated data. CAESAR with independent Gaussian likelihood used with and without temporal filtering beforehand (red and blue respectively), CAESAR with temporal GP likelihood (green) and the functional connectivity model proposed in [11] (yellow). Accuracy of cluster assignments is measured using adjusted mutual information (AMI; top row). Accuracy of the cluster timecourse estimates is measured using root mean squared error (RMSE; bottom row). Accuracy was measured as a function number of nodes *N* (column 1), number of clusters *K* (column 2), number of time-points *T* (column 3), and noise level *σ* (column 4) while keeping all other variables fixed and simulating five different data sets for each combination. The shaded areas illustrate the minimum and maximum performance among these random data sets.

To examine the robustness of CAESAR thoroughly, we repeated the above described simulation process five times with four different data generation conditions. The different conditions were generated by varying *N*, *K*, *T*, and *σ* one at a time while keeping all other variables fixed. The accuracies of the cluster and timecourse estimates for each condition are summarized in the columns of Fig 4. Accuracy of cluster assignments is measured with adjusted mutual information (AMI) [29], which is scaled so that one corresponds to perfect reconstruction and zero corresponds to the trivial solution where all nodes are put in the same cluster. Accuracy of the cluster timecourse estimates is measured using root mean squared error (RMSE).

The first column of Fig 4 shows that the IT-model clearly fails when the average number of nodes is too small for a given SNR and data is not filtered. In contrast, temporal filtering, either beforehand or within the model, results in perfect cluster reconstructions using our timecourse based models. Notably, performance of the connectivity-based model declines on either end of the spectrum, suggesting parameter sensitivity. In all experiments the variance of the noisy FMRI data was scaled to one, which means that the noise level $\sigma =0.949=\sqrt{0.9}$ corresponds to a SNR of 0.1/0.9. This setting is already quite challenging, but we see this as fairly realistic, as our experiments with real FMRI data showed similar noise estimates with the same model assumptions. As can be expected, decreasing the number of nodes *N* with fixed *K*, results in less accurate cluster timecourse estimates in all cases, as fewer node timecourse observations are obtained from each cluster. In terms of timecourse reconstruction, using the GP model on unfiltered data appears to be slightly better than filtering beforehand. The second column of Fig 4 illustrates the same behaviour from a slightly different viewpoint as *K* is increased while *N* and SNR are kept fixed. In either case, i.e. when varying *N* or *K* while keeping the other fixed, the salient change is actually the number of nodes per cluster.

The third column of Fig 4 demonstrates that the GP-model remains very stable at different data lengths *T* and is able to recover (near) perfect cluster assignments for all chosen values of *T*. The independent model, on the other hand, cannot properly combine the information across different time points and fails to recover the correct cluster structure with all settings except when operating on filtered data, in which case performance is on par with the GP-model. The same holds for the connectivity-based model. Notably, the timecourse estimation of the prefiltered data is worse than that of the GP model under low to moderate noise levels. We have also examined the effect of TR, by fixing either the number of samples or the time span. These results are presented in S1 Fig and demonstrate a small, TR-dependent advantage of the GP-model in reconstructing timecourses and cluster recovery.

Finally, the fourth column of Fig 4 shows that, on its own, the IT-model fails to learn the cluster structure when *σ* becomes too large, i.e., when the SNR gets too low. Filtering helps a great deal here as well, as all models are able to achieve perfect cluster reconstructions at all but the highest noise levels.

#### Running time

**Fig 5:**
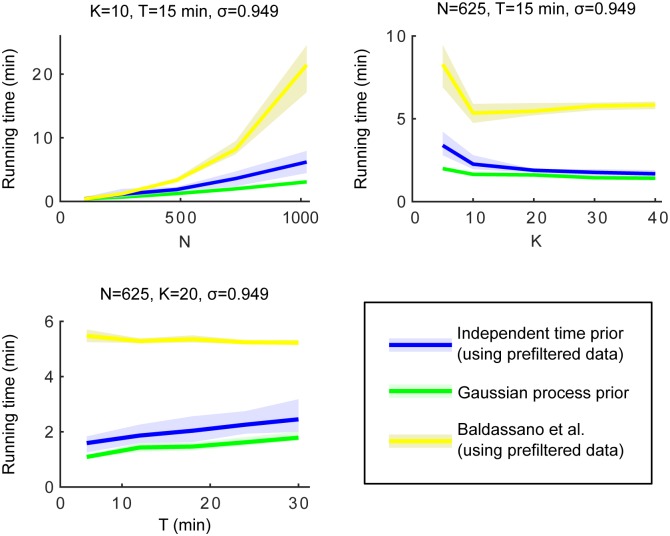
Empirical running times of dd-CRP models. Independent Gaussian priors (IT-model; blue), time-dependent GP priors (GP-model; green) and the connectivity-based model. All models were applied to the same simulated data sets as in the first three columns of Fig 4.

Fig 5 shows the running times for the different data generation conditions. The fourth condition is not shown, because running time is unaffected by SNR. Note that exactly the same number of posterior samples were drawn with all conditions, hence these figures also illustrate per-sample scaling of the proposed approach.

The first panel of Fig 5 shows that computational burden increases approximately linearly with the dimensionality of the clustering problem with both timecourse priors. This linearity is due to the truncation of the decay function, i.e. a hard spatial constraint, as this limits the possible link assignments to a fixed number, regardless of the total number of nodes.

The second panel of Fig 5 shows that, with fixed *N*, increasing the number of clusters *K* also slightly increases computational costs. This is probably due to the fact that as *K* increases, average cluster size decreases, which leads to fewer “internal” nodes. When updating a node, the number of required cluster-likelihood evaluations is equal to the number of clusters in the neighbourhood (including the node itself) after removing that node’s link. Hence, nodes on the borders of clusters are more costly to update. In addition, decreased cluster size also results in more of them potentially being present in a border node’s neighbourhood.

The third panel of Fig 5 shows that inference with the GP-based model gets slower as *T* increases, and from theory we know that pre-computing all the required auxiliary variables defined in S1 Inference scales as $O(T^{3})$. However, since these variables need to be updated only once for each GP hyperparameter configuration, the practical speed of our batch method is very close to the independent model with typically sized FMRI data sets. The accuracy comparisons from the third column of Fig 4 also suggest that inference with the GP-based model could possibly be sped up by restricting to an interesting segment of the actual measurement to reduce the number of data points.

### Resting state FMRI

**Fig 6:**
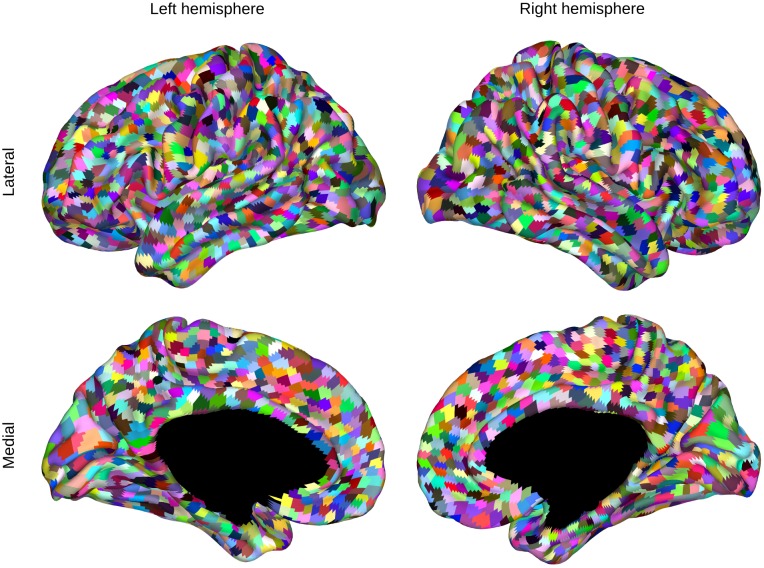
Group parcellation based on 20 participants.

**Fig 7:**
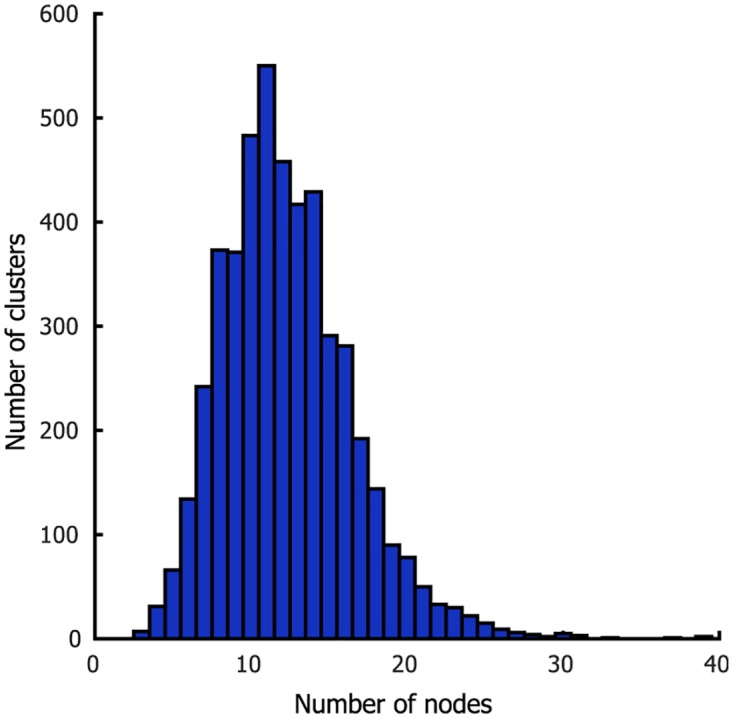
Distribution of cluster sizes in the 20-participant parcellation.

Next, CAESAR was applied to rsFMRI data using the population MC framework. Data from the first run of the group of 20 participants were used to obtain a group parcellation. For each hemisphere, the sample with the highest importance weight served as the maximum a posteriori (MAP) parcellation estimate. The resulting parcellation is shown in Fig 6 and contains 2391 and 2429 clusters for left and right hemisphere respectively. The distribution of the number of nodes in a cluster is shown in Fig 7. Note that there are no singleton clusters, the smallest cluster consisted of three nodes and 95% of clusters contained at least 7 nodes. Performing a second level clustering, i.e. using these clusters and their timecourses as nodes in a second application of CAESAR, resulted in little or no further clustering.

**Fig 8:**
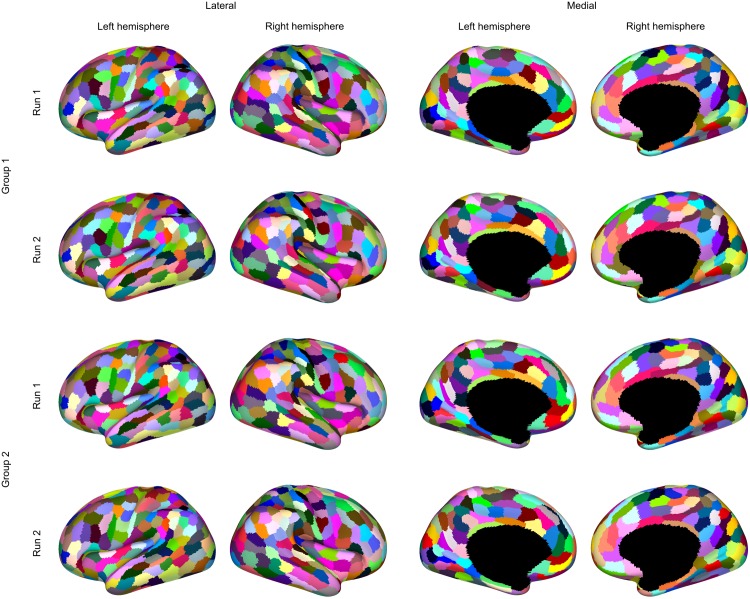
Group parcellations with a soft constraint on minimum cluster size. The first and second row visualise the parcellation based on runs 1 and 2 respectively for group 1. Similarly for the third and fourth rows w.r.t. group 2.

**Fig 9:**
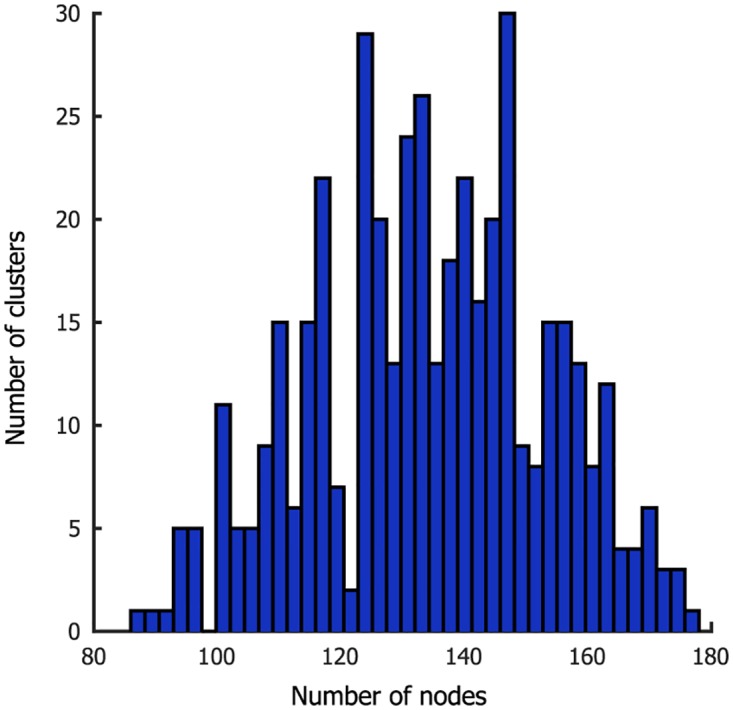
Distribution of cluster sizes in the size-constrained parcellations.

Using the cluster-size prior, we obtain a more manageable 220 and 224 clusters in the left and right cortical hemispheres respectively, with the parcellations shown in Fig 8. The cluster-size distribution, shown in Fig 9, demonstrates the soft constraint on cluster sizes. The model is still free enough to settle on a variety of clusters.

As the cluster-size prior resulted in a manageable number of clusters, we focused on examining reproducibility and generalisability by applying CAESAR, with these settings, to the remaining three datasets. Over the four datasets the number of clusters ranged from 217 to 220 for the left hemisphere and 211 to 224 for the right. While the number of clusters found was quite consistent, that alone does not say anything about reproducibility of the structure. This was therefore assessed by computing the AMI between all pairs of parcellation estimates based on each of the four datasets. For comparison, we used spatially-constrained Ward-clustering on each of the datasets and cut the trees to match the number of cluster that CAESAR found for that dataset. The average values for within and between group AMI for Ward-clustering and CAESAR are presented in Table 1. Across all comparisons, CAESAR consistently scored higher than Ward.

**Table 1:** Reproducibility, as measured with AMI.

	Within group		Between group	
	Ward	CAESAR	Ward	CAESAR
Left	0.76	0.82	0.76	0.80
Right	0.76	0.82	0.75	0.80
Overall	0.76	0.82	0.75	0.80

Within-group similarity is the average AMI between parcellations of pairs of runs within groups. Between-group similarity is the average AMI between parcellations of pairs of runs across groups.

Another measure of performance is the amount of variance explained by the cluster timecourses. Because the models were applied filtered and unfiltered data, we looked at variance explained in the unfiltered data. Timecourses for the Ward-clustering were obtained by taking the mean of filtered voxel-timecourses. For CAESAR, we used the group-level parcellations obtained with the cluster-size prior. Timecourses were estimated with the IT-model on pre-filtered data and the GP-model on unfiltered data, while holding the parcellation fixed. By using the IT-model on filtered data, we can get an idea of the effect of the parcellation itself. Any improvement by the GP-model beyond this can then be attributed to the timecourse estimation itself.

The mean explained-variances are presented in Table 2. These results show that not only is our model better at explaining the data that was used for the parcellation, it also generalises considerably better. Although the IT-model consistently explained more variance than Ward-clustering, the major improvement comes from the use of the GP-model in estimating timecourses.

**Table 2:** Percentage of variance explained.

		CAESAR	
	Ward	IT-model	GP-model
Within run			
Left	9.50%	9.72%	11.58%
Right	9.47%	9.69%	11.57%
Overall	9.49%	9.70%	11.58%
Within group			
Left	9.37%	9.56%	11.40%
Right	9.34%	9.52%	11.39%
Overall	9.36%	9.54%	11.40%
Between run			
Left	9.29%	9.45%	11.29%
Right	9.25%	9.41%	11.27%
Overall	9.27%	9.43%	11.28%

Within-run explained-variance is the average over datasets of variance explained by the parcellation obtained from that dataset. Within-group explained-variance is the average over pairs of runs within groups where the parcellation based on one dataset is used explain variance in the other. Between-group explained-variance is the average over pairs of runs across groups.

## Discussion

As the simulations show, the GP-model is a marked improvement over the IT-model in terms of both the parcellation obtained and the quality of time-course reconstruction. Although similar performance can obtained by temporal filtering beforehand, the GP-model appears to be slightly more robust at estimating timecourses, especially in the case of more favourable SNRs. This is probably due to the GP-model utilising information from all voxels in a cluster and it becomes more salient as TR increases. Importantly, as running times show, this comes at virtually no cost in computational time.

Applied to the FMRI data, this model shows that the HCP data is rich enough to support a fine-grained parcellation. Attempting a second level parcellation, i.e., use the cluster timecourses as input to the GP-model, resulted in mostly singleton clusters. This suggests that the large number of clusters is not due to variations in SNR. If this were the case, *Z*-scoring the estimated cluster timecourses would correct for this and allow for more mergers in the second-level clustering. This high-resolution parcellation might be a useful way to perform data reduction, especially given the high-quality of estimated timecourses, while maintaining some level of spatial specificity.

The cluster-size prior may be viewed as a step back, as the point is to estimate the number clusters from the data. The necessity of this compromise is illustrated in the extremely large number of clusters obtained without such a prior. Even worse, tweaking the parameters of the GP-prior significantly affects the parcellation estimate. A similar effect can be seen in [11], where they manipulate the number of clusters using a parameter of the likelihood function. These parameter tweaks destroy interpretability in terms of the assumptions that are made. We decided to include a prior on cluster sizes, because this offers the user a clearly interpretable dial to turn. The soft constraint is still an improvement over fixing the number of clusters, as we can still estimate the number of clusters. Moreover, we would argue that setting the scale, i.e. the size of clusters, of the desired parcellation is what one is trying to achieve by selecting the number of clusters and our cluster-size prior is a more direct way of doing this.

In their simulations, [11] showed superior performance for their model as compared to other approaches, including local similarity [9] and Ward clustering. In terms of robustness to noise, our approach performs slightly better than that of [11], although it should be noted that the GP-model operates on unfiltered data. In terms of parameter sensitivity, CAESAR is considerably more robust.

The model proposed in [11] is related to CAESAR in that they have the same prior on partitions. An important difference is that their approach clusters connectivity profiles, a popular tactic in the parcellation literature. The advantage of operating on connectivity, as opposed to the underlying timecourses themselves, is that group analyses can easily be performed simply by averaging connectivity. We chose to model the timecourses themselves, because our objective is not only to provide a parcellation, but also to provide the corresponding functional signal, which can be used in a secondary analysis.

Operating on the connectivity matrix means that computational cost is independent of the number of timepoints, whereas the cost for our model scales linearly in that regard. On the other hand, when clustering the connectivity matrix, sweeps over the link assignments in **λ** scale cubically in the number of nodes, as opposed to linear scaling when clustering timecourses. Hence, in many situations our model would be faster. In theory, CAESAR could also be applied to correlation profiles (preferably after a Fisher transformation), which would replace the linear scaling in both *N* and *T* with quadratic scaling in *N*, or less if one only considers a subset of the profile.

Parameter sensitivity is an important aspect of any model. In the simulations, CAESAR’s performance, given theoretically-justified parameter-choices, is quite consistent regardless of the data conditions. This is contrasted by a sensitivity to number of nodes per cluster of the connectivity-based model. On the other hand, GP parameter choices do influence the number of clusters returned for empirical data. A possible future extension might include placing a prior on the parameters of the covariance function, or on the function itself [30], to circumvent the strong influence of these parameters. Nevertheless, these parameters have a clear enough interpretation that we can justify their choice.

Spatially-constrained Ward-clustering has been shown to be the best among several of the most commonly used parcellation approaches [28]. While the improvements over prefiltering are modest in the simulations, the improvements over spatially-constrained Ward-clustering are considerable in terms both variance explained and reproducibility on empirical data. Strikingly, while CAESAR’s parcellation estimate on its own increased the explained variance by about 2% (0.19 ± 0.03 percentage points), additionally employing the GP-model to estimate the posterior timecourse resulted in a 22% (2.05 ± 0.04 percentage points) increase of explained variance. This illustrates the gain in the quality of timecourses when using CAESAR.

In this work we used Gibbs sampling in a population MC framework to perform posterior inference. This approach requires sampling several chains in parallel for each hemisphere, which results in long running times. CAESAR could benefit greatly from alternative forms of posterior inference that would speed up the process, Variational Bayes, which uses approximate distributions in order to speed up the search for the posterior mode, is such an alternative. While this technique is generally used in parametric models, non-parametric applications have also been developed [31, 32]. However, [31] is not applicable to ddCRPs and [32] is only applicable to sequential ddCRPs (models where the order of the nodes matter), with no clear way of generalizing to non-sequential ddCRPs.

CAESAR represents a principled approach to parcellate whole-brain FMRI data and obtain high quality time-courses for the constituent clusters. The parcellations are highly reproducible and generalisable, even given a modest amount of data. While not pursued in this paper, the probabilistic nature of CAESAR also enables the propagation of uncertainty in parcellation, as well as timecourses, to connectivity estimates and beyond [33, 34].

## Supporting Information

S1 Inference This supplementary information describes the inference procedure in more detail. (PDF)

S1 Code This supplementary information contains a Matlab implementation of CAESAR. (ZIP)

S1 Fig Parcellation- and timecourse-recovery accuracy as a function of TR. (PDF)
