# Supplementary material for: Let’s Not Waste Time: Using Temporal Information in Clustered Activity Estimation with Spatial Adjacency Restrictions (CAESAR) for Parcellating FMRI Data
Source: PLoS One. 2016 Dec 9;11(12):e0164703. doi: 10.1371/journal.pone.0164703 (PMC5147788; doi:10.1371/journal.pone.0164703)
Supplement: S1 Inference — (PDF) [file pone.0164703.s001.pdf]

1 Inference

1.1 Posterior Inference Using Single-Chain Monte Carlo

Combining the observation model with the priors for the cluster time-series and the link assignments (Eqs. 2, 3 and 7 in the main text), we can write the joint posterior distribution as

$$p(\mathbf{X}, \boldsymbol{\lambda}, \boldsymbol{\theta}|\mathbf{Y}, \mathbf{A}) = Z^{-1} \prod_{k=1}^{K} \Big(p(\mathbf{Y}_k|\mathbf{x}_k, \boldsymbol{\phi}, \tau, \boldsymbol{\lambda}) p(\mathbf{x}_k|\boldsymbol{\sigma}_{\mathrm{m}}^2, \boldsymbol{l}) \Big) p(\boldsymbol{\lambda}|\mathbf{A}) p(\boldsymbol{\theta}), \qquad (1)$$

where $Z = p(\mathbf{Y}|\mathbf{A})$ is the normalization term, all the hyperparameters have been denoted with $\boldsymbol{\theta} = \{\boldsymbol{\phi}, \tau, \boldsymbol{\sigma}_{\mathrm{m}}^2, \boldsymbol{l}\}$, and $\mathbf{Y}_k$ is shorthand for all the node time series belonging to cluster k given some partition $\boldsymbol{\pi}(\boldsymbol{\lambda})$. Because the posterior distribution (1) is analytically intractable, meaning that we cannot analytically compute the normalization constant Z or the expectations of any of the unknown variables, we form a sample-based approximation using MCMC methods.

To draw samples from the posterior we construct a partially collapsed Gibbs sampling framework, which integrates over the T-dimensional cluster time series $\mathbf{x}_k$ when possible. At each iteration of the sampling algorithm we repeat the following steps:

1. Draw the GP hyperparameters $\boldsymbol{\psi} = [\log(\boldsymbol{\sigma}_{\mathrm{m}}^2), \log(\boldsymbol{l})]$ from their marginal distribution that is obtained by integrating over the cluster time series $\{\mathbf{x}_k\}_{k=1}^K$ as described in Appendix 1.3:

$$\begin{aligned} \boldsymbol{\psi} &\sim p(\boldsymbol{\psi}|\mathbf{Y}, \boldsymbol{\lambda}, \boldsymbol{\phi}, \tau) \\ &\propto \prod_{k=1}^{K} \Big(p(\mathbf{Y}_k|\boldsymbol{\lambda}, \boldsymbol{\phi}, \tau, \boldsymbol{\psi}) \Big) p(\boldsymbol{\psi}). \end{aligned} \qquad (2)$$

   Because the conditional posterior (2) is not analytically tractable, we use slice sampling to draw from the unnormalized density. If separate hyperparameters are assigned to each cluster, (2) can be factorized and the sampling can be done independently for each $k = 1, ..., K$.

2. Draw the cluster time series $\mathbf{X} = [\mathbf{x}_1, ..., \mathbf{x}_K]^{\mathrm{T}}$ given the current link assignments $\boldsymbol{\lambda}$ and the hyperparameters:

$$\mathbf{x}_k \sim p(\mathbf{x}_k|\mathbf{Y}, \boldsymbol{\lambda}, \boldsymbol{\phi}, \tau, \boldsymbol{\psi}), \qquad (3)$$

   which can be done independently for $k = 1, ..., K$ as described in Appendix 1.3. The time series $\mathbf{X}$ are used only as auxiliary variables that facilitate subsequent sampling steps for the noise hyperparameters $\boldsymbol{\phi}$ and τ.

3. Draw the overall noise precision τ given the cluster time series $\mathbf{x}_k$ and the time-specific noise parameters $\boldsymbol{\phi}$:

$$\tau \sim p(\tau|\mathbf{Y}, \mathbf{X}, \boldsymbol{\phi}) \propto p(\mathbf{Y}|\mathbf{X}, \tau, \boldsymbol{\phi}) p(\tau). \qquad (4)$$

The conditional posterior (4) is a Gamma distribution from which samples can be drawn directly.

4. Draw the time-specific noise parameters $\boldsymbol{\phi} = [\phi_1, ..., \phi_T]$ given the cluster time series $\mathbf{x}_k$ and the overall noise precision τ:

$$\begin{aligned}\phi_t &\sim p(\phi_t|\mathbf{y}_t, \mathbf{x}_t, \tau) \\ &\propto \prod_{n=1}^{N} \mathcal{N}(y_{n,t}|x_{\pi_n,t}, (\tau\phi_t)^{-1})p(\phi_t) \end{aligned} \tag{5}$$

for $t = 1, ..., T$, where $\mathbf{y}_t$ and $\mathbf{x}_t$ denote the observation and cluster time series vectors for each t, respectively. Each of the conditional posteriors in (5) is a Gamma distribution form which samples can be drawn directly.

5. Draw the node link assignments by doing a (number of) Gibbs sweep(s) over all the nodes $i = 1, ..., N$ given the hyperparameters:

$$\begin{aligned}\lambda_i &\sim p(\lambda_i|\boldsymbol{\lambda}_{-i}, \mathbf{Y}, \boldsymbol{\phi}, \tau, \boldsymbol{\psi}) \\ &\propto \prod_{k=1}^{K} \Big(p(\mathbf{Y}_k|\boldsymbol{\lambda}, \boldsymbol{\phi}, \tau, \boldsymbol{\psi})\Big)p(\boldsymbol{\lambda}|\mathbf{A}). \end{aligned} \tag{6}$$

The marginal density $p(\mathbf{Y}_k|\boldsymbol{\lambda}, \boldsymbol{\phi}, \tau, \boldsymbol{\psi})$ can be computed as described in Appendix 1.3.

In theory, the collapsed step 5 should be done before steps 2-4, but here we have moved it to the last position because we wish to record samples $\{\boldsymbol{\lambda}^{(l)}, \boldsymbol{\phi}^{(l)}, \tau^{(l)}, \boldsymbol{\psi}^{(l)}\}$, where the node link assignments $\boldsymbol{\lambda}$ represent the current hyperparameter setting. It is also better to draw the overall noise precision in step 3 before the time-specific noise parameters. This way τ will capture the average noise level and ϕ_t will remain on average around one representing time-specific adjustments to τ. Clearly, the majority of the algorithm's running time is spent in step 5, because it can contain thousands of individual link assignments, each of which can require marginal likelihood computations related to one cluster split proposal and several different cluster merge proposals.

1.2 Parallel Inference Using Population Monte Carlo

Because typical FMRI measurements contain a large number of voxels and the measurements can be noisy, several different cluster assignment configurations can explain the observed data with relatively high posterior densities. Consequently, the posterior distribution (1) can have multiple distinct modes corresponding to different values of $\boldsymbol{\lambda}$. Therefore, a single Gibbs sampling chain can get stuck in some low density modes for a large number of iterations resulting in slow overall convergence. In contrast, with fixed $\boldsymbol{\lambda}$, the algorithm of Section 1.1 typically converges quickly.

To speed-up convergence, we propose to utilize several parallel chains in a population Monte Carlo (PMC) framework [1]. At each step of the PMC

algorithm, we run one iteration of the Gibbs sampler of Section 1.1 in J parallel chains that propagate one sample particle each. Next we combine the resulting particles into a single posterior approximation using an importance sampling resampling update. First, the particles are initialized, e.g., by drawing random values for the $\boldsymbol{\lambda}^{(j,0)}$ from the dd-CRP prior and choosing appropriate values for the hyperparameters $\boldsymbol{\phi}^{(j,0)}$, $\tau^{(j,0)}$, and $\boldsymbol{\psi}^{(j,0)}$. Then, at each iteration i, the algorithm draws a sample from the proposal distributions $q(\cdot)$ in parallel for each particle $j = 1, ..., J$:

$$
\begin{aligned}
\boldsymbol{\psi}^{(j,i)} &\sim q_1(\boldsymbol{\psi}) \\
&= p(\boldsymbol{\psi}|\boldsymbol{\lambda}^{(j,i-1)}, \boldsymbol{\phi}^{(j,i-1)}, \tau^{(j,i-1)}) \\
\mathbf{x}_k^{(j,i)} &\sim q_2(\mathbf{x}_k) \\
&= p(\mathbf{x}_k|\boldsymbol{\lambda}^{(j,i-1)}, \boldsymbol{\phi}^{(j,i-1)}, \tau^{(j,i-1)}, \boldsymbol{\psi}^{(j,i)}) \\
&\qquad\qquad\qquad\qquad \text{for } k = 1, ..., K \\
\tau^{(j,i)} &\sim q_3(\tau) \\
&= p(\tau|\mathbf{X}^{(j,i)}, \boldsymbol{\phi}^{(j,i-1)}) \\
\phi_t^{(j,i)} &\sim q_4(\phi_t) \\
&= p(\phi_t|\mathbf{x}_t^{(j,i)}, \tau^{(j,i)}) \quad \text{for } t = 1, ..., T \\
\lambda_n^{(j,i)} &\sim q_5(\lambda_n) \\
&= p(\lambda_n|\boldsymbol{\lambda}_{1:n-1}^{(j,i)}, \boldsymbol{\lambda}_{n+1:N}^{(j,i-1)}, \boldsymbol{\phi}^{(j,i)}, \tau^{(j,i)}, \boldsymbol{\psi}^{(j,i)}) \\
&\qquad\qquad\qquad\qquad \text{for } n = 1, ..., N.
\end{aligned}
$$

Which is followed by computing the importance weight for each particle $j = 1, ..., J$ as

$$
w_{j,i} \propto \frac{p(\mathbf{Y}|\boldsymbol{\lambda}^{(j,i)}, \tau^{(j,i)}, \boldsymbol{\phi}^{(j,i)}, \boldsymbol{\psi}^{(j,i)})}{q_1(\boldsymbol{\psi}^{(j,i)}) \prod_k q_2(\mathbf{x}_k^{(j,i)}) q_3(\tau^{(j,i)})} \frac{p(\boldsymbol{\lambda}^{(j,i)}|\mathbf{A}) p(\boldsymbol{\theta}^{(j,i)})}{\prod_t q_4(\phi_t^{(j,i)}) \prod_n q_5(\lambda_n^{(j,i)})} \tag{7}
$$

and resampling particles using the normalized weights. After resampling, the particles are given equal importance weights and typically only the descendants of the best particles survive to the next iteration.

After sufficient number of iterations, the posterior expectations of any function $h(\cdot)$ of the unknowns can be summarized using a suitable number of iterations from the end of the PMC chain as:

$$
\mathrm{E}\Big(h(\boldsymbol{\lambda}, \boldsymbol{\phi}, \tau, \boldsymbol{\psi})\Big) \approx \frac{\displaystyle\sum_{i=i_1}^{i_2} \sum_{j=1}^{J} w_{j,i} h(\boldsymbol{\lambda}^{(j,i)}, \boldsymbol{\phi}^{(j,i)}, \tau^{(j,i)}, \boldsymbol{\psi}^{(j,i)})}{i_2 - i_1}. \tag{8}
$$

Because resampling is done at each iteration i, the weights can be set to equal values: $w_{j,i} = 1/J$. Compared to a single Gibbs chain, the PMC algorithm has much better chances to escape local modes, because at each iteration the J parallel Gibbs sweeps explore more efficiently the high-dimensional posterior distribution near the particles that have survived from the previous iterations.

1.3 Batch GP Inference

This appendix describes a batch method for computing the marginal likelihood $p(\mathbf{Y}|\boldsymbol{\lambda}, \boldsymbol{\theta})$ and the conditional posterior $p(\mathbf{X}|\mathbf{Y}, \boldsymbol{\lambda}, \boldsymbol{\theta})$, which is a crucial part of the Gibbs sampler of Appendix 1.1. The method is very efficient for a small to medium number of time points, roughly $T \in (0, 10000]$ depending on memory constraints.

Because the GP prior (Eq. 3 in the main text) is defined separately for each cluster time course $\mathbf{x}_k$ and the likelihood (Eq. 2 in the main text) can also be factored over k, we can do the inference separately for each cluster. The conditional posterior distribution of $\mathbf{x}_k$ given all hyperparameters and $\boldsymbol{\lambda}$, is Gaussian and it is given by

$$\begin{aligned} p(\mathbf{x}_k|\mathbf{Y}, \boldsymbol{\theta}) &= Z_k^{-1} p(\mathbf{Y}_k|\mathbf{x}_k, \boldsymbol{\lambda}, \boldsymbol{\theta}) p(\mathbf{x}_k|\boldsymbol{\theta}) \\ &= \mathcal{N}(\boldsymbol{\mu}_k, \boldsymbol{\Sigma}_k), \end{aligned} \tag{9}$$

where $\mathbf{Y}_k$ is a $N_k \times T$ matrix containing the node measurements from cluster k. The posterior mean $\boldsymbol{\mu}_k$ and the covariance $\boldsymbol{\Sigma}_k$ and the marginal likelihood $Z_k = p(\mathbf{Y}_k|\boldsymbol{\lambda}, \boldsymbol{\theta}) = \int p(\mathbf{Y}_k|\mathbf{x}_k, \boldsymbol{\lambda}, \boldsymbol{\theta}) p(\mathbf{x}_k|\boldsymbol{\theta}) d\mathbf{x}_k$ can be written as

$$\begin{aligned} \boldsymbol{\mu}_k &= \left(\mathbf{B}^{\mathrm{T}} \boldsymbol{\Sigma}_\epsilon^{-1} \mathbf{B} + \mathbf{K}^{-1}\right)^{-1} \mathbf{B}^{\mathrm{T}} \boldsymbol{\Sigma}_\epsilon^{-1} \mathbf{y}_k \\ \boldsymbol{\Sigma}_k &= \left(\mathbf{B}^{\mathrm{T}} \boldsymbol{\Sigma}_\epsilon^{-1} \mathbf{B} + \mathbf{K}^{-1}\right)^{-1} \\ \log Z_k &= -\frac{T N_k}{2} \log(2\pi) - \frac{1}{2} \log |\mathbf{K}||\boldsymbol{\Sigma}_\epsilon||\boldsymbol{\Sigma}_k^{-1}| \\ &\quad - \frac{1}{2} \mathbf{y}_k^{\mathrm{T}} \left(\boldsymbol{\Sigma}_\epsilon^{-1} - \boldsymbol{\Sigma}_\epsilon^{-1} \mathbf{B} \boldsymbol{\Sigma}_k \mathbf{B}^{\mathrm{T}} \boldsymbol{\Sigma}_\epsilon^{-1}\right) \mathbf{y}_k, \end{aligned}$$

where $\mathbf{y}_k = \mathrm{vec}(\mathbf{Y}_k)$ is formed by stacking the columns of $\mathbf{Y}_k$ into a single vector, $\mathbf{B} = \mathbf{I}_T \otimes \mathbf{1}_{N_k}$, $\boldsymbol{\Sigma}_\epsilon = \mathrm{diag}(\tau^{-1}\phi_1^{-1}, ..., \tau^{-1}\phi_T^{-1}) \otimes \mathbf{I}_{N_k}$, $\mathbf{1}_{N_k}$ is a $N_k \times 1$ vector of ones, and $\otimes$ denotes the Kronecker product. In the Gibbs sampling for $\boldsymbol{\lambda}$, the marginal likelihood Z_k needs to be evaluated for each proposed cluster configuration and it requires a Cholesky decomposition of the $T \times T$ matrix $\boldsymbol{\Sigma}_k^{-1} = \mathbf{B}^{\mathrm{T}} \boldsymbol{\Sigma}_\epsilon^{-1} \mathbf{B} + \mathbf{K}^{-1}$, which is computationally expensive.

Using the properties of the Kronecker product we can show that the marginal likelihood can be evaluated very efficiently if we pre-compute the following auxiliary variables after each hyperparameter update:

$$\begin{aligned} \tilde{\mathbf{Y}} &= \mathbf{Y} \mathrm{diag}(\tau^{1/2}\phi_1^{1/2}, ..., \tau^{1/2}\phi_T^{1/2}) \\ \tilde{\mathbf{B}} &= \mathrm{diag}(\tau^{1/2}\phi_1^{1/2}, ..., \tau^{1/2}\phi_T^{1/2}) \mathbf{K}^{1/2} \\ \mathbf{U} \boldsymbol{\Lambda} \mathbf{U}^T &= \tilde{\mathbf{B}}^{\mathrm{T}} \tilde{\mathbf{B}} \quad \text{eigendecomposition of a } T \times T \text{ matrix} \\ \mathbf{V} &= \tilde{\mathbf{Y}} \tilde{\mathbf{B}} \mathbf{U} \\ \mathbf{w} &= (\tilde{\mathbf{Y}} \circ \tilde{\mathbf{Y}}) \mathbf{1}_T \\ C_0 &= -\frac{T N_k}{2} \log(2\pi) + \frac{N_k}{2} \sum_{t=1}^{T} \log(\tau \phi_t), \end{aligned}$$

where $\circ$ is the entry-wise product. We assume that $\mathbf{K}^{1/2}$ needs to be computed only after updating the GP hyperparameters. At each new cluster proposal we can evaluate the marginal likelihood as

$$\log Z_k = -\frac{1}{2}\sum_{t=1}^{T}\left(\log(d_{k,t}) - \frac{(\sum_{\pi_n=k}\mathbf{V}_{n,t})^2}{d_{k,t}}\right) - \frac{1}{2}\sum_{\pi_n=k}\mathbf{w}_n + C_0,$$

where $d_{k,t} = 1 + \lambda_t N_k$ and which involves only scalar summations over $n \in \{n|\pi_n = k\}$ and $t = 1, ..., T$.

In step 2 of the Gibbs sampler of Appendix 1.1 we need to draw time courses from the conditional posterior and after convergence we also want to compute the conditional mean and covariance of $\mathbf{x}_k$ for summarizing the final results. These can be obtained using

$$\begin{aligned}
\boldsymbol{\mu}_k &= \mathbf{L}_1\left([d_1^{-1}, ..., d_T^{-1}] \circ \sum_{\pi_n=k}\mathbf{V}_{n,:}\right)^{\mathrm{T}} \\
\boldsymbol{\Sigma}_k &= \mathbf{L}_1\mathrm{diag}(d_1^{-1}, ..., d_T^{-1})\mathbf{L}_1^{\mathrm{T}} \\
\mathbf{x}_k &= \boldsymbol{\mu}_k + \mathbf{L}_2\left([d_1^{-1/2}, ..., d_T^{-1/2}]^{\mathrm{T}} \circ (\mathbf{L}_2^{\mathrm{T}}\mathbf{r})\right),
\end{aligned}$$

where $\mathbf{L}_1 = \mathbf{K}^{1/2}\mathbf{U}$, $\mathbf{L}_2 = \mathbf{K}^{1/4}\mathbf{U}$ and $\mathbf{r}_t \sim \mathcal{N}(0, \mathbf{I}_T)$. Here we assume that $\mathbf{L}_1$ and $\mathbf{L}_2$ are obtained using the previously computed auxiliary variables and the eigendecomposition of the prior covariance $\mathbf{K}$.

References

[1] O. Capp, A. Guillin, J. M. Marin, C. P. Robert, Population Monte Carlo, Journal of Computational and Graphical Statistics 13 (4) (2004) 907–929. URL `http://www.jstor.org/stable/27594084`
