# Supplementary material for: Let’s Not Waste Time: Using Temporal Information in Clustered Activity Estimation with Spatial Adjacency Restrictions (CAESAR) for Parcellating FMRI Data
Source: PLoS One. 2016 Dec 9;11(12):e0164703. doi: 10.1371/journal.pone.0164703 (PMC5147788; doi:10.1371/journal.pone.0164703)
Supplement: S1 Fig — (PDF) [file pone.0164703.s003.pdf]

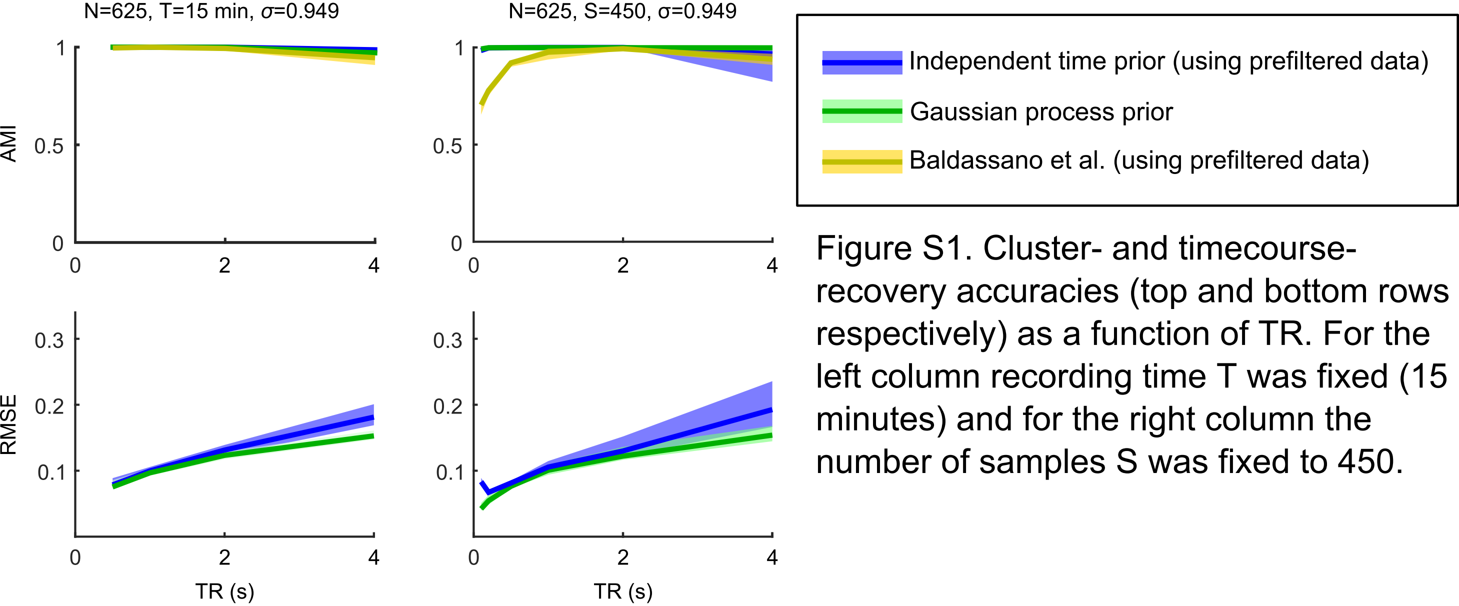

Figure S1. Cluster- and timecourse-recovery accuracies (top and bottom rows respectively) as a function of TR. For the left column recording time T was fixed (15 minutes) and for the right column the number of samples S was fixed to 450.
